# Coexistence of lung adenocarcinoma and pulmonary tuberculosis within a single lesion: A rare case report

**DOI:** 10.1097/MD.0000000000017378

**Published:** 2019-09-27

**Authors:** Hong Gwon Byun, Jin Young Yoo, Sung Jin Kim, Ok Jun Lee, Min Young Yoo

**Affiliations:** a Department of Radiology, Chungbuk National University Hospital; b Department of Radiology; c Department of Pathology, Chungbuk National University College of Medicine; d Department of Nuclear Medicine, Chungbuk National University Hospital, Cheongju, Korea.

**Keywords:** computed tomography, lung cancer, pulmonary tuberculosis

## Abstract

**Rationale:**

Pulmonary tuberculosis and lung adenocarcinoma are highly prevalent pulmonary diseases associated with high mortality. However, the coexistence of lung cancer and pulmonary tuberculosis is rare. Further, the morphological features of lung cancer with coexisting pulmonary tuberculosis are similar to that of lung cancer without pulmonary tuberculosis, even though the lesion is predominantly cavity. For these reasons, the diagnosis in patients with coexisting lung cancer and pulmonary tuberculosis could be delayed until the advanced stage, and therefore, prognosis in these patients is worse compared with that of lung cancer patients without coexisting pulmonary tuberculosis. Therefore, early diagnosis of the condition is essential for initiating timely and suitable treatment.

**Patient concerns:**

A 67-year-old man was detected abnormal finding on chest CT performed outside the hospital during health screening without significant symptom.

**Diagnoses:**

Chest CT revealed a 3.2, irregular, enhancing cavitary mass in right lower lobe of lung and PET-CT revealed significant uptake of 18 FDG by the cavitary mass, which was suggestive of lung cancer. Pathology results confirmed a diagnosis of coexisting lung adenocarcinoma and tuberculosis.

**Interventions and outcome:**

The patient underwent a right lower lobectomy. No significant complications occurred in a 24 month post-surgery follow-up period

**Lessons:**

Although rare, the coexistence of lung adenocarcinoma and tuberculosis within a single lesion can occur. Therefore, early diagnosis of such a lesion is essential to improve the prognosis in affected patients.

## 1 Introduction

Tuberculosis is one of the leading causes of death due to infectious diseases, and it is a global health threat. Most tuberculosis infections are pulmonary tuberculosis. Further, the mortality rate among pulmonary tuberculosis patients is 7% to 35%.^[1]^ Lung cancer is one of the leading causes of cancer deaths around the world with a mortality rate of 29%.^[2]^ Although pulmonary tuberculosis and lung cancer have high prevalence rates, they rarely occur together in the same lesion. According to a study conducted by the National Cancer Institute, the incidence rate of pulmonary tuberculosis coexisting with lung cancer, which typically occurs in the upper lobe, was estimated to be under 2%. Moreover, patients with pulmonary tuberculosis are at high risk of lung cancer, and men with tuberculosis are estimated to have a 2-fold increase in the risk of lung cancer.^[3]^ However, there has been no case report on the coexistence of lung cancer and tuberculosis within a single lesion in the lower lobe. Here, we report a case with computed tomography (CT) findings of adenocarcinoma and pulmonary tuberculosis coexisting within a single lesion in the lower lobe and correlate with pathologic findings. The patient has provided informed consent for publication of the case.

## 2 Case presentation

**Figure 1:**
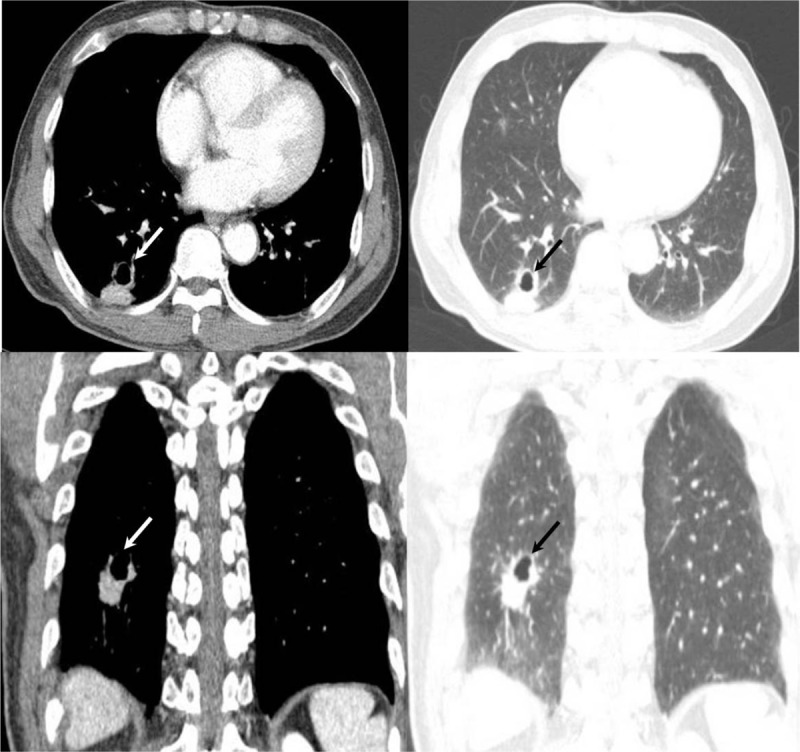
(A–D) Initial chest CT on health screening revealed a 3.2 cm cavitary lesion in the right lower lobe that consisted of an area of consolidation and cavity (white arrow indicated cavitary mass in RLL on mediastinal window setting, black arrow indicated cavitary mass in RLL on lung window setting).

**Figure 2:**
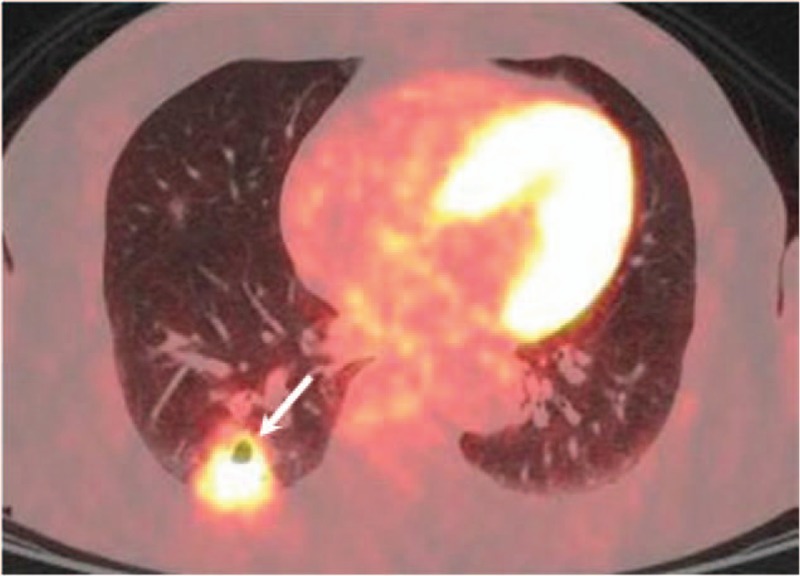
PET-CT image showed showing a significant uptake of 18F-fluorodeoxyglucose by the cavitary mass (SUVmax = 8.3).

A 67-year-old man was referred to our hospital due to abnormal finding on chest CT performed outside the hospital during health screening. He had no significant symptom. He was a former smoker with 100 pack years. All laboratory findings were within normal limits. CT scan performed outside the hospital revealed a 3.2 cm, irregular, enhancing cavitary mass, which consisted of cavity and consolidation within a single lesion in the right lower lobe (Fig. 1A–D). There was no evidence of mediastinal lymph node enlargement on the chest CT. The patient was admitted to our hospital to investigate the pulmonary lesion. Positron emission tomography-computed tomography (PET-CT) revealed significant uptake of 18F-fluorodeoxyglucose (FDG) by the cavitary mass (SUVmax = 8.3), which indicated a probability of lung cancer. There was no significant difference in FDG uptake between the cavity and the area of consolidation within the lesion (Fig. 2).

**Figure 3:**
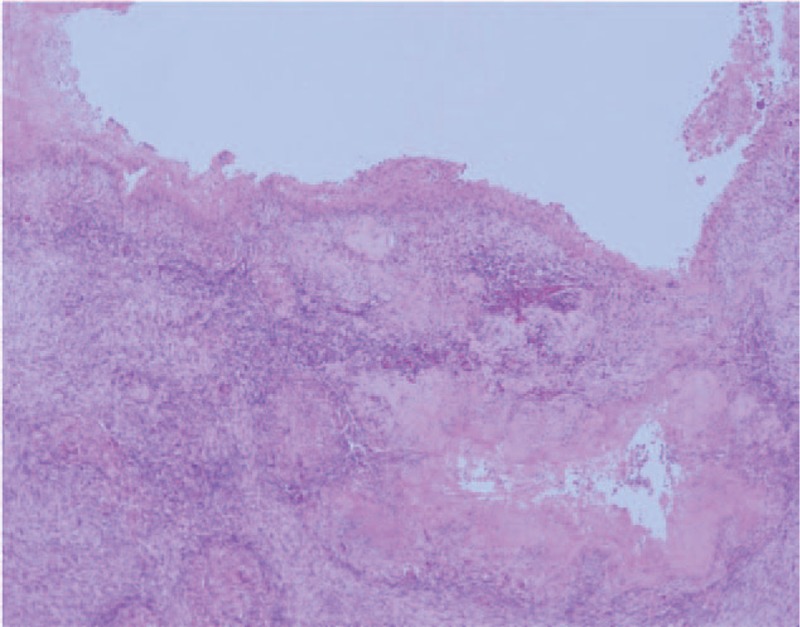
Microscopic findings; multiple areas of caseous necrosis with glands in the cavity and a few tumor cells; most tumor cells are highly concentrated at the borderline of the cancerous lesion and tuberculosis cavity (Hematoxylin-Eosin stain, ×40).

The patient underwent a right lower lobectomy. As shown in the CT image, the lesion revealed areas of cavity and consolidation. Pathologic results showed that adenocarcinoma (moderately differentiated) and pulmonary tuberculosis coexisted within the lesion (Fig. 3). The consolidation was confirmed as due to adenocarcinoma, and the cavity was confirmed as due to pulmonary tuberculosis. In addition, there was evidence of adenocarcinoma at the borderline between the consolidation and cavity. After AFB staining, the pulmonary tuberculosis was confirmed as active tuberculosis.

The size of the lung adenocarcinoma was 2 cm and lymph node metastasis was absent. Thus, a diagnosis of stage Ib lung cancer was made. A recent study showed that adjuvant chemotherapy is not recommended for routine use in patients with stage Ib lung cancer after complete resection,^[4]^ and therefore, we did not perform adjuvant chemotherapy. Additionally, the patient had active pulmonary tuberculosis within the same mass. Usually after surgery for pulmonary tuberculosis, post-operative anti-tuberculosis medication is necessary for at least 12 months.^[5,6]^ Accordingly, our patient was administrated anti-tuberculosis therapy for 12 months. No complications associated with the surgery or tuberculosis infection occurred in the 24 month follow-up period.

## 3 Discussion

Most lung cancers that coexist with pulmonary tuberculosis are large lobulated masses with mediastinal lymph node enlargement.^[7]^ Moreover, the morphological features of lung cancer with coexisting pulmonary tuberculosis are similar to that of lung cancer without pulmonary tuberculosis, and if the lesion is predominantly cavitary lesion, the diagnosis is delayed.^[7]^ Coexistence of pulmonary tuberculosis and adenocarcinoma is rare, and the pathogenesis of coexisting tuberculosis and lung cancer remains unknown.^[7]^ There are several hypotheses reported. First, the tumor could arise from a previous pulmonary tuberculosis lesion, which is called scar cancer. Tuberculosis can cause persistent inflammation resulting in fibrosis, scarring, and host-tissue damage. Fibrosis from old tuberculosis lesions can cause lymphadenopathy and enhance the accumulation of carcinogens in the area.^[2,3,8]^ In addition, lung scarring, resulting from a favorable environment of spontaneous cure after repeated pulmonary tuberculosis infection, favors tumor growth. Moreover, pulmonary parenchyma can promote atypical epithelial cell proliferation and metaplasia in the healed cavity.^[2,8]^ Second, tuberculosis mycobacterial cell wall components can induce nitric oxide production and reactive oxygen species, both of which are involved in carcinogenesis.^[2]^ Chronic tuberculosis infections lead to secretion of DNA-damaging reactive oxygen and nitrogen species by tuberculosis-infected macrophages, which also produce the most powerful member of the epidermal growth factor family, which plays an important role as a paracrine growth factor early in the process of carcinogenesis.^[2]^ Third, carcinoma may reactivate pulmonary tuberculosis. The onset of lung cancer in the area of inactive pulmonary tuberculosis stimulates reactivation of *Mycobacterium tuberculosis*. In addition, the association between primary lung cancer and pulmonary tuberculosis may be related to an increase in opportunistic infections resulting in reactivation of tuberculosis in cancer patients.^[7]^

In prior studies, the prognosis of patients with coexisting lung adenocarcinoma and pulmonary tuberculosis has been controversial.^[9]^ However, a recent study showed that hazard ratio and mortality of coexisting lung adenocarcinoma and pulmonary tuberculosis were significantly higher than without pulmonary tuberculosis. This is due to the possibility of either lesion (lung adenocarcinoma or tuberculosis) masking the other one.^[10]^ A standard treatment plan for coexisting lung adenocarcinoma and pulmonary tuberculosis has not been established. However, many studies have shown that when the physician cannot absolutely exclude lung adenocarcinoma, surgical treatment is the treatment of choice followed by anti-tuberculosis medication and adjuvant chemotherapy according to the pathologic staging.^[4,5,6,9,10]^

Our patient had a 3.2 cm, irregular, enhancing cavitary mass in the right lower lobe. We considered this lesion as lung cancer initially because lung cancer could have a cavitary form, and there were no other findings suggestive of pulmonary tuberculosis infection on CT scan. Further, coexisting lung cancer and pulmonary tuberculosis have a very low incidence. In addition, PET-CT revealed that this was a highly FDG uptaken lesion. PET-CT cannot distinguish active pulmonary tuberculosis and adenocarcinoma because both are highly FDG uptaken lesions. Therefore, we recognized this lesion was lung cancer. However, the lesion consisted of 2 parts: a 1.6 cm area of consolidation and a cavity of 1.6 cm size. The 2 parts were abutting to each other side by side. Pathologic findings confirmed the area of consolidation as adenocarcinoma and cavity as pulmonary tuberculosis. In addition, there was evidence of adenocarcinoma at the borderline of the 2 parts. As mentioned above, pulmonary tuberculosis has several characteristics that could lead to lung cancer. In our patient, there were no old pulmonary scar lesion or other findings suggestive of active tuberculosis in both lungs on chest CT. Furthermore, there was an increase in the number of adenocarcinoma cells at the borderline of the 2 parts. Therefore, the pathogenesis of this lesion is presumed to be the second hypothesis. However, the limitation was that it was not possible to determine the chronological order of appearance of the diseases in this patient.

In conclusion, we presented a rare case of coexistence of lung adenocarcinoma and tuberculosis within a single lesion. Patients who initially present with active tuberculosis and lung cancer have lower survival rates than those presenting with lung cancer without tuberculosis. Therefore, in cases where a cavitary mass in the lung is revealed in a CT image, we should consider the possibility of lung cancer coexisting with pulmonary tuberculosis and appropriately select the treatment and management strategy.

## Author contributions

**Writing – original draft:** Hong Gwon Byun, Jin Young Yoo, Sung Jin Kim, Ok Jun Lee, Min Young Yoo.
